# Genetic source tracking of human plague cases in Inner Mongolia-Beijing, 2019

**DOI:** 10.1371/journal.pntd.0009558

**Published:** 2021-08-03

**Authors:** Jianyun Li, Yumeng Wang, Fang Liu, Xiaona Shen, Yiting Wang, Mengguang Fan, Yao Peng, Shuyi Wang, Yilan Feng, Wen Zhang, Yanning Lv, Huijuan Zhang, Xin Lu, Enmin Zhang, Jianchun Wei, Lijuan Chen, Biao Kan, Zhongbing Zhang, Jianguo Xu, Wenrui Wang, Wei Li

**Affiliations:** 1 General Center for Disease Control and Prevention of Inner Mongolia Autonomous Region, Huhehot, China; 2 National Institute for Communicable Disease Control and Prevention (ICDC), China CDC, Changping, Beijing, China; 3 State Key Laboratory of Infectious Disease Prevention and Control, Changping, Beijing, China; 4 Beijing Center for Disease Control and Prevention, Beijing, China; Institut Pasteur, FRANCE

## Abstract

On 12 November 2019, one couple from the Sonid Left Qi (County) in the Inner Mongolia Autonomous Region was diagnosed with pneumonic plague in Beijing. The wife acquired the infection from her husband. Thereafter, two bubonic plague cases were identified in Inner Mongolia on November 16^th^ and 24^th^. In this study, genome-wide single nucleotide polymorphism (SNP) analysis was used to identify the phylogenetic relationship of *Yersinia pestis* strains isolated in Inner Mongolia. Strains isolated from reservoirs in 2018 and 2019 in Inner Mongolia, together with the strain isolated from Patient C, were further clustered into 2.MED3m, and two novel lineages (2.MED3q, 2.MED3r) in the 2.MED3 population. According to the analysis of PCR-based molecular subtyping methods, such as the MLVA 14 scheme and seven SNP allele sequencing, Patients A/B and D were classified as 2.MED3m. In addition, strains from rodents living near the patients’ residences were clustered into the same lineage as patients. Such observations indicated that human plague cases originated from local reservoirs. Corresponding phylogenetic analysis also indicated that rodent plague strains in different areas in Inner Mongolia belong to different epizootics rather than being caused by spreading from the same epizootic in *Meriones unguiculatus* in 2019.

## Author summary

The plague still poses a significant threat to human health, such as in Madagascar, which experienced a large pneumonic plague outbreak in 2017. The pneumonic plague always arouses great concern for its pathogenicity and propagation, especially in densely populated cities. In November 2019, one couple in the Inner Mongolia Autonomous Region suffered pneumonic plague and was transferred to Beijing. Subsequently, another two bubonic plague cases were identified in Inner Mongolia. Genome-wide SNP analysis, together with two PCR-based molecular subtying methods, was used for source-tracking purposes. Corresponding results indicated that human plague cases originated from local rodent plague epizootics. Because there were no obvious geographic barriers between the plague foci area in Inner Mongolia and its counterpart area in Mongolia, early warning and risk communication is necessary.

## Introduction

Plague is an acute infectious disease caused by *Yersinia pestis*. Plague killed millions of individuals in Europe in the 14^th^ century, and the third plague pandemic caused tens of thousands of deaths in China and the world in the 19^th^ and 20^th^ centuries[1]. Plague is mainly a disease of wild rodents, and their parasitic fleas are transmitting vectors. Three major clinical plague types exist in humans: bubonic, pneumonic and septicaemic plague. Person-to-person transmission typically occurs only among pneumonic plague patients.

There are four plague foci in the Inner Mongolia Autonomous Region (Inner Mongolia), i.e., the *Meriones unguiculatus* plague focus in the Inner Mongolian Plateau (focus I, note: the nomenclature of natural plague foci in China is consistent with previous literature [2]), the *Microtus brandti* plague focus in the Xilin Gol Grassland (L), the *Spermophilus dauricus* plague focus in the Song-Liao Plain (H) and the *Marmota sibirica* plague focus in the Hulun Buir Plateau (N, silent plague focus[3]). The Inner Mongolia Plateau *M*.*unguiculatus* focus was first identified in 1954. The total area of this focus is 134,803 km^2^ and encompasses 22 counties/Qis (another designation, equivalent to county) in the central and western parts of Inner Mongolia [4]. The primary host in the Inner Mongolia Plateau *M*.*unguiculatus* plague focus is *M*.*unguiculatus*. In addition, *Spermophilus dauricus*, *Meriones meridianus*, *Arvicola lureus* and *Spermophilus erythrogenys* also play important roles in maintaining the enzootic persistence of the plague in this focus. The main vectors are *Nosopsyllus laeviceps*, *Xenopsylla conformis* and *Neopsylla pleskei* [4].

A total of five human plague outbreaks occurred in Inner Mongolia in the first half of the twenty century, i.e., the Manzhouli pneumonic plague outbreaks occurred in 1910 and 1920, pneumonic and bubonic plague outbreaks occurred in western areas in Inner Mongolia in 1917 and 1928, and the Tongliao area plague occurred in 1947 [4]. Human plague has been well controlled since 1959 in Inner Mongolia. During 1960–2018, only nine human plague cases with two deaths occurred in Inner Mongolia, and the most recent human plague case was reported in 2004 due to skinning a dead hare [5]. The *Y*.*pestis* pathogens in the Inner Mongolia Plateau *M*.*unguiculatus* plague natural focus are biovar mediaevalis [6, 7]. Human plague infection always occurred after plague epizootics in *M*.*unguiculatus* on the Inner Mongolia Plateau. In the last six years, the plague epizootics of *M*.*unguiculatus* have been active again, especially in 2018 and 2019 (Chinese national plague surveillance data, 2018 and 2019).

There were a total of four human plague cases reported in Inner Mongolia and Beijing in 2019. The index case (patient A) was a 43-year-old male herdsman. He presented clinical symptoms on October 25^th^, and his wife (patient B, 46 years old) developed infection when she accompanied and cared for patient A. The couple was treated in local hospitals in Inner Mongolia, and the condition further deteriorated. Then, they were transferred to a general hospital in Beijing and admitted to the intensive care unit (ICU). On November ^12th^, the two cases were identified as pneumonic plague cases based on real-time PCR and immunology methods by the Beijing CDC and China CDC. This is the first pneumonic plague imported into a large city, such as Beijing, since the founding of the People’s Republic of China [8]. Thereafter, two bubonic plague cases were identified in Inner Mongolia on November 14^th^ and 24^th^.

In this study, molecular epidemiology methods, together with geographic information, were used to identify the infection source of the four patients. In addition, the phylogenetic relationships of rodent plague epidemics also illustrated that different epizootics existed among *M*.*unguiculatus* in Inner Mongolia in 2019.

## Methods

### Ethics statement

All procedures involved in the survey and diagnosis of human plague cases were performed in accordance with ethical standards. Corresponding sampling in epidemiological investigation or treatment obtained patients’ verbal consent, and the procedure was approved by the Review Board of Ethics at the National Institute for Communicable Disease Control and Prevention, China CDC. All procedures performed in this study were in accordance with the ethical standards of the national research committee.

### Epidemiological investigation of human plague cases in Inner Mongolia and Beijing

Epidemiological information was obtained from standard plague case survey forms recorded by epidemiological professionals in the Inner Mongolia CDC or Beijing CDC, as well as official survey reports by the China CDC. Clinical symptoms and treatment processes were recorded from epidemiological reports by the local CDC. The patient treatment information was derived from the description of the doctor’s records. The diagnostic standards used in this plague outbreak included the identification of *Y*.*pestis* isolates by culture with a special phage lysis assay[9] or PCR positivity together with immunological methods positive for F1 antigen in clinical samples, i.e., reverse indirect haemagglutination assay (RIHA) and/or colloidal gold-immunochromatography assay positive. The serum samples collected from patients were tested for antibodies against the F1 antigen via indirect haemagglutination assay (IHA) [10]. The PCR method in this study was developed by members of our team in ICDC[11], including the *caf*1 gene and YPO0392 segment target, and the sensitivity and specificity of two targets of PCR were evaluated.

### DNA preparation from strains and clinical specimens

In 2018 and 2019, a total of 18 and 117 *Y*.*pestis* isolates were obtained, respectively, in routine rodent plague surveillance by the General Center for Disease Control and Prevention of Inner Mongolia Autonomous Region. We selected research strains based on which they were isolated from different points (villages) or different resources or from different months in 2018 or in 2019, so these strains could represent corresponding strains collected in Inner Mongolia in 2018 and 2019. A total of forty *Y*.*pestis* strains isolated from Inner Mongolia were included in this study (S1 Table), including five strains isolated from *M*.*unguiculatus* or their parasitic fleas in 2018 and thirty-five strains in 2019 (S1 Table). The strain from patient C (2019.LF) isolated by the ICDC. Genomic DNA of each bacterium was extracted according to the manual of the Qiagen DNA Mini Kit (QIAGEN) in the Biosafety Level 3 (BSL-3) Laboratory of the ICDC. Because no *Y*.*pestis* strain was cultured from patients A, B, and D (they were administered antibiotics before sampling), DNA templates from clinical specimens (Patient A/B: blood and sputum; Patient D: lymph node aspirates) were extracted by a DNeasy Blood & Tissue Kit (QIAGEN) according to the manufacturer’s instructions.

### Phylogenetic analyses based on whole genomic SNPs

A total of 105 genomes were used to construct the phylogenetic relationship of *Y*.*pestis* strains (S1 Table) according to genome-wide SNP analysis methods described previously [1, 6], including those of 40 *Y*.*pestis* strains isolated from Inner Mongolia in this study, 11 *Y*.*pestis* genome sequences representing the global diversity of *Y*.*pestis* species, and fifty-four *Y*.*pestis* genome sequences in GenBank isolated from Inner Mongolia or other provinces in China previously. The whole genomic DNA samples in this study were sequenced using the Illumina HiSeq 2000 platform (Illumina, San Diego, CA). A maximum likelihood tree was constructed by the concatenated SNP using MEGA 6.0 software [12] rooted with *Y*.*pseudotuberculosis* strain IP32953 (accession number NZ_CP009712.1) [6, 13]. The nomenclature of phylogenetic lineages in this study follows previous literature [6, 13], and novel lineages were designated in succession.

### Phylogenetic position of patients by multiple-locus variable number of tandem repeat (VNTR) analysis (MLVA) and SNP analysis

Because no *Y*.*pestis* isolates were obtained from clinical specimens of patients A, B and D and by whole genome sequencing of clinical specimens (patient A and B: blood and sputum, patient D: lymph node aspirates), only limited pieces of *Y*.*pestis* sequences were identified. For example, the DNA sequence read output associated with *Y*.*pestis* in Patient D’s lymph node aspirates only accounted for approximately 3000 reads. Thus, it is not possible to obtain all SNP information for phylogenetic analysis through such sequencing data. PCR-based molecular subtyping methods, including MLVA and SNP locus sequencing analysis, were used to identify the phylogenetic population position of patients A, B, and D in the phylogenetic tree. For those sequenced *Y*.*pestis* strains from surveillance reservoirs and strain 2019.LF from patient C, the number of tandem repeat profiles of 14 VNTR loci were obtained by finding primers for VNTRs in genomic sequences and identifying the corresponding number in the corresponding VNTR locus [14]. These profiles of 14 VNTRs were compared with corresponding VNTR profiles from patients A, B and D.

To further identify the associated lineage of patients A, B and D, we compared and identified seven SNP loci [6] that present polymorphic characteristics in the 2.MED3 among *Y*.*pestis* strains isolated in Inner Mongolia in 2018 and 2019 (S1, S2 and S4 Tables). The seven SNP loci were identified by PCR and sequencing, with their primers listed in S3 Table. PCR was performed in 50-μl reaction mixtures containing 5 μl of buffer with Takara Taq DNA polymerase (10×), 3 μl of the purified genomic DNA of clinical samples, 0.5 μM each oligonucleotide primer, and an appropriate volume of sterile water. Referencing the known phylogenetic position of *Y*.*pestis* strains isolated from reservoirs in 2019, we combined the patient’s pathogen into the same lineage with the same canonical SNPs in Inner Mongolia (S3 Table).

## Results

### Details of epidemiology and treatment of human plague cases in Inner Mongolia and Beijing

There were a total of four human plague cases reported in Inner Mongolia and Beijing in 2019. According to epidemiological investigation records, there were no epidemiological connections among patients A/B (considered one event), patient C and patient D.

Patient A was a 43-year-old male herdsman who lived in Bayannaoer Village, Sonid Left Qi (county), Xilingol League (prefecture) in Inner Mongolia. On October 25^th^, after digging the soil on his farm, he presented with the onset of coughing and vomiting with fever (39.2°C). In the following days, his cough was aggravated with blood-tinged sputum, chills, chest pain and breathlessness. The patient sought treatment in local county hospitals on October 27^th^ and was administered antibiotics (levofloxacin and azithromycin). On October 31^st^, his wife (patient B, 46 years old) suffered similar symptoms after she accompanied and cared for patient A. Patient B was treated with antibiotics (levofloxacin and azithromycin) in the local hospital from October 31^st^ to November 3^rd^ but did not show significant improvement. Then, the couple was transferred to a general hospital in Beijing by ambulance; thereafter, they were admitted to the ICU and treated with antibiotics (cefoperazone sodium, sulbactam sodium, moxifloxacin) for another eight days. By genome sequencing of alveolar lavage fluid specimens from the two patients, limited pieces of *Y*.*pestis* sequences were found in these specimens, and such results led the clinical doctors to began to suspect the two patients might suffer pneumonic plague and reported the two cases to the Beijing CDC on November 11^th^. The two patients were subsequently confirmed to have pneumonic plague on November 12^th^ by real-time PCR for blood and sputum specimens of patients A and B, together with positivity by the colloidal gold-immunochromatography assay and RIHA targeting the F1 antigen for sputum specimens. In addition, the F1 antibody results (both samples were collected on November 11^th^, 2019) by the IHA method were positive for the two patients, with titers of the F1 antibody of 1:320 (patient A) and 1:640 (patient B). Thereafter, the two patients were transferred to a special infectious disease hospital in Beijing, where the index pneumonic plague case (patient A) was cured with combination treatment (gentamicin and levofloxacin). However, patient B unfortunately died of multiple organ failure.

On November ^11th^, 2019, patient C (55-year-old male), who originally lived and worked in a mine in Bayentala Village, Xianghuang Qi, Xilingol League, sought treatment in a hospital in Huade County of Ulanqab City for left axillary lymph nodes swollen with high fever (39.4°C). The laboratory in the China CDC identified *Y*.*pestis* by culture and real-time PCR methods from lymph node aspirates. Patient C had an exposing history of skinning dead hare (on November 5^th^) and was diagnosed with bubonic plague on November ^13th^. Patient C was cured after 17 days by administration of antibiotics (streptomycin, meropenem, and moxifloxacin).

The fourth case (patient D) was a left inguinal lymphadenitis patient (48-year-old female, herdswoman) in Siziwang Qi in Ulanqab City. She was diagnosed with bubonic plague on November 24^th^ for typical clinical manifestations, with a positive PCR assay result on lymph node aspirates. From the onset of fever symptoms to the time point that she was diagnosed with bubonic plague, patient D used various antibiotics (amoxicillin, levofloxacin, doxycycline, and ceftazidime) by herself or by a local hospital, so no *Y*.*pestis* was successfully cultured from lymph node aspirates. The patient also presented an F1 antibody fourfold increase by IHA. Patient D was finally cured by administration of streptomycin, together with levofloxacin, doxycycline, and ceftazidime.

### Phylogenetic relationship of *Y*.*pestis* in Inner Mongolia

**Fig 1:**
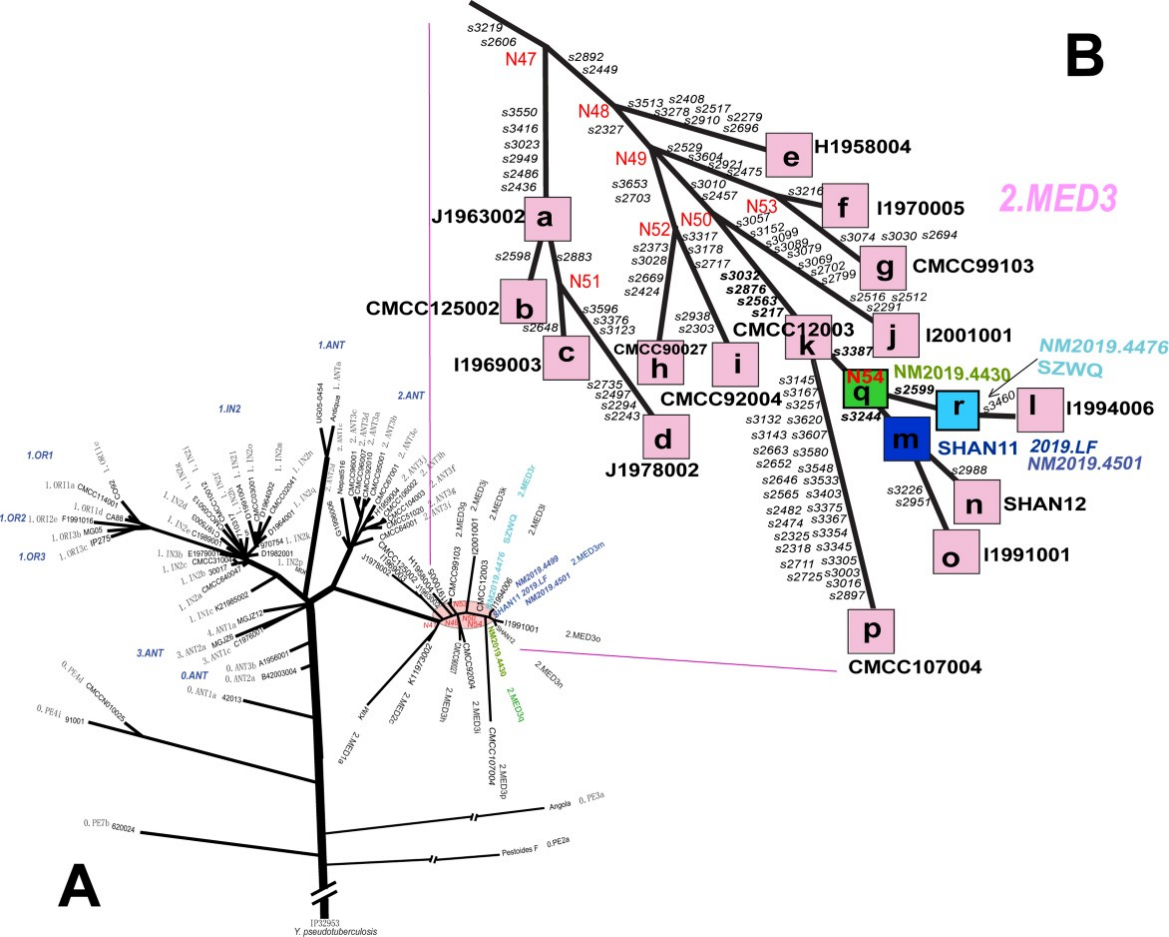
Phylogenetic tree of *Y*.*pestis* and the lineage relationships in 2.MED3 population. A: Phylogenetic relationships rooted in *Y*. *pseudotuberculosis* (IP32953) were determined by the maximum likelihood method based on genomic SNPs, which included 40 *Y*.*pestis* strains in this study and 65 genomic sequences of *Y*.*pestis* from GenBank. Note: The length of the branch in Fig 1A does not reflect the SNP polymorphic frequency; B: The phylogenetic position and lineages of *Y*.*pestis* in 2.MED3 population in this study.

**Fig 2:**
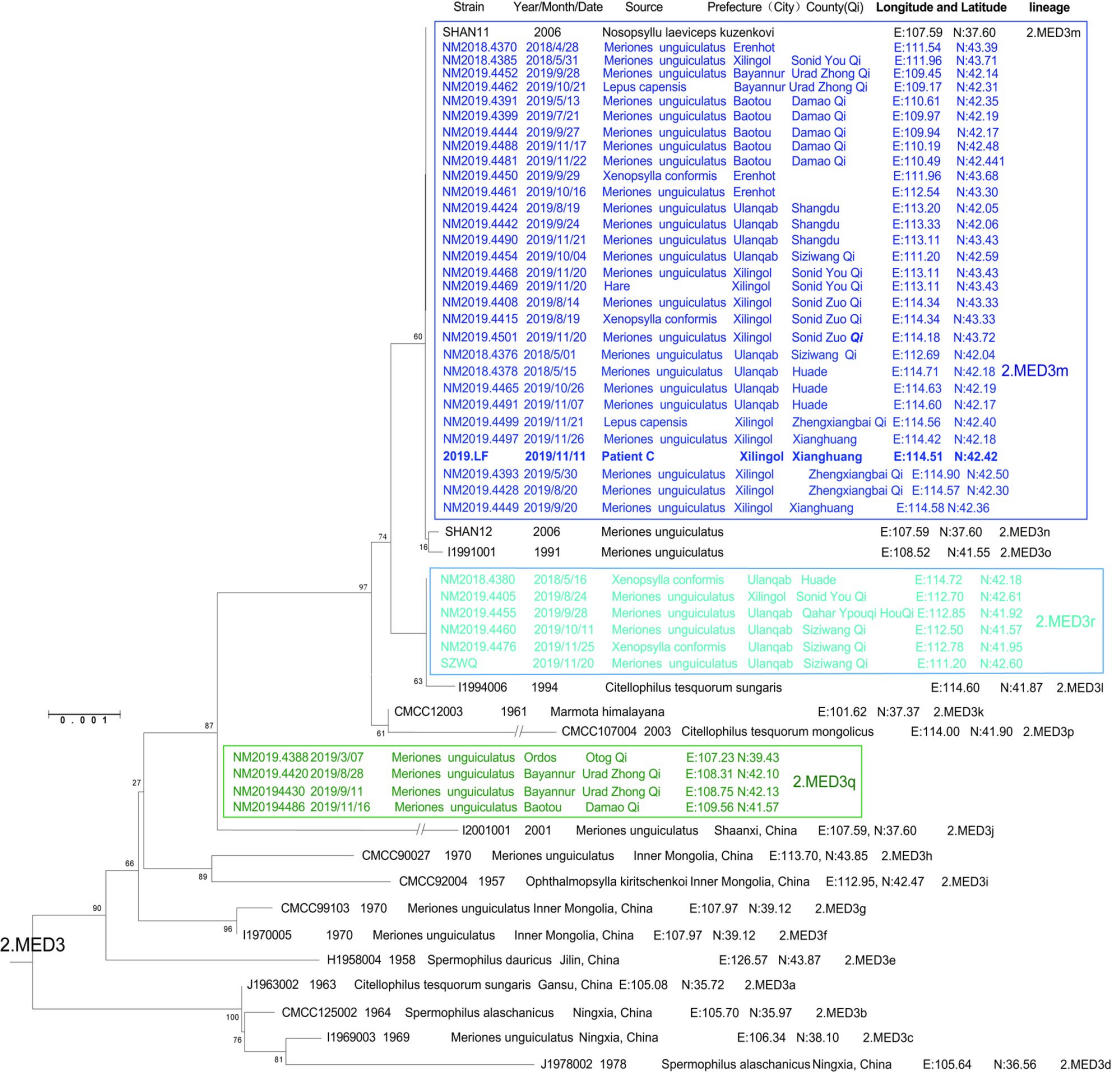
Dendrogram of phylogenetic lineages in the 2.MED3 population. The phylogenetic structure and corresponding lineages in the 2.MED3 population was determined from the SNP assignments by the maximum likelihood method.

As shown in Fig 1A, the 2019.LF strain (patient C) isolated from Xianghuang Qi, together with all strains isolated from reservoirs in 2018 and 2019 in Inner Mongolia, was clustered into 2.MED3 population in the phylogenetic tree. Those previously sequenced strains in 2.MED3 population were designated into various lineages (2.MED3a~p) (Figs 1 and 2, *Y*.*pestis* strains isolated from Inner Mongolia or adjacent provinces such as Shanxi, Hebei, Ningxia in China)[6] While, *Y*.*pestis* strains isolated in 2018 and 2019 in Inner Mongolia could be divided into three lineages (2.MED3m, 2.MED3q, 2.MED3r), in which the 2.MED3q, 2.MED3r belonged to novel lineages (Figs 1 and 2 and S2 and S4 Tables). Because SNP s3387 was used to differentiated 2.MED3k to N54, and the ancestral and derived nucleotide in allele of SNP s3387 is A to G [6], one of interesting observations is that 2.MED3q coincidently located in the connection node N54 (Fig 1B).

Referring to the result in previous research that the MLVA 14 VNTR scheme was considered to have a corresponding discriminatory power compatible with SNP-based phylogenetic analysis at the population level[14], the MLVA 14 scheme was used to illustrate the genomic relationship among DNAs of *Y*.*pestis*-associated strains in patients A, B and D’ specimens. The profiles of 14VNTRs of patients A/B and D were the same as those of local rodent surveillance strains, while strains from local hosts were clustered into 2.MED3 lineages (S4 Table). Therefore, we deduced that the phylogenetic position of patients A/B and D should also be 2.MED3 population in the genome-wide SNP phylogenetic tree.

### Source tracking of human plague patients in Inner Mongolia and in Beijing

Strains isolated from reservoirs in Inner Mongolia in 2018 and 2019 were grouped into three lineages, i.e. 2.MED3m, 2.MED3q, 2.MED3r in the phylogenetic tree (Figs 1 and 2). The strain isolated from patient C was clustered into 2.MED3m lineage. Using sequencing data of seven SNP loci (primers in S3 Table), we classified the pathogens’ DNA from patients A/B and D into lineage 2.MED3m and 2.MED3r, respectively (S4 Table).

Patient A was a shepherd, and the family of patients A/B lived in an independent house on semidesert grassland. Before patient A suffered pneumonic plague, he had an exposure history of digging soil in a local plague natural focus, where *M*.*unguiculatus* serves as a primary plague host. From the retrospective investigation, patient A denied once biting by fleas or contacting any dead rodents or animals before he became infected. Patient A was suspected to be infected by inhaling infectious aerosols originating from the decayed bodies of reservoirs in nets or in burrows. In the process of the enhanced rodent surveillance campaign, a strain (NM2019.4501) was isolated from *M*.*unguiculatus* five hundred metres away from the house of patients A/B, and the strain was also grouped into lineage 2.MED3m. The profiles of 14VNTRs of patients A/B were also same with profiles in local rodent strains. In addition, a number of strains isolated from dead rodents in the same county were clustered into lineage 2.MED3m (Figs 2 and S1). These observations indicate that serious *M*.*unguiculatus* plague epizootics existed in the local area and that the infection of patient A should have originated from a local plague epizootic.

For patient C, the infection was acquired by skinning a dead hare he found in the field. There were no epidemiological connections between patients A/B and patient C. Slaughtering or skinning a sick and dead host animal (such as hare) is one of infection way in Inner Mongolia *M*.*unguiculatus* plague natural focus, and the primary clinical manifestation, corresponding to the way and site of infection, frequently includes lymphadenopathy or septicemia[4]. The strain from patient C was also grouped into lineage 2.MED3m. Unfortunately, the hare that patient C skinned did not remain for testing. However, some strains isolated from locally dead *M*.*unguiculatus* (NM2019.4449, NM2019.4497) in Xianghuang Qi were grouped into lineage 2.MED3m, while a strain (NM2019.4499) isolated from *Lepus capensis* in an adjacent county (Zhengxiangbai Qi) was also identified as 2.MED3m (Figs 2 and S1). Such observations inferred that the human plague was transmitted from a diseased hare, while the hare plague originated from *M*.*unguiculatus*.

Patient D was a shepherd, and she suffered left inguinal bubonic plague when herding sheep on an *M*.*unguiculatus* plague natural focus. One *Y*.*pestis* strain (SZWQ) isolated from *M*.*unguiculatus* in the same village of patient D was identified as 2.MED3r, the same as the deduced lineage for Patient D (Fig 2). Thus, patient D was suspected to acquire infection by flea biting, and the corresponding clinical symptoms also supported this inference.

### Epizootics of plague in 2018 and 2019 in Inner Mongolia

The primary host in the desert or semidesert steppe of the Inner Mongolia Plateau is *M*.*unguiculatus*, and the life span of *M*.*unguiculatus* is 1 to 2 years, so one obvious feature for the population of *M*.*unguiculatus* is that the density of this type host changed greatly in certain years. In 2019, a much higher density of the major host in this focus was observed. For example, the overall density of *M*.*unguiculatus* was 4.23 per square hectare in 2019, while the number was 3.04 and 2.76 in 2018 and 2017, respectively. In particular, the density of *M*.*unguiculatus* in some counties in 2019 was much higher than the historical average level (2/hm^2^) on the Inner Mongolia Plateau [4], such as Otog Qi (12.77/hm^2^), Huade County (11.58/hm^2^), Hangjin Qi (8.25/hm^2^), Urad Qian Qi (7.81/hm^2^), Sonid You Qi (4.85/hm^2^), Otog Qian Qi (4.85/hm^2^), Sonid Zuo Qi (4.25/hm^2^), Zheng xiang bai Qi (4.14/hm^2^), Damao Qi (3.81/hm^2^), and Urad Qian Qi (3.71/hm^2^) (Chinese National Plague Surveillance Data, 2019). Correspondingly, more reservoirs were captured in 2019 than in 2018. For example, a total of 10698 rodents were captured and autopsied in 2019, while the number was 5534 in 2018. In addition, more *Y*.*pestis* isolates (n = 118) were collected from rodent plague surveillance in 2019 than in 2018 (n = 18). The geographical distribution of strains in this study together with their phylogenetic lineages in 2019 is shown in S1 Fig. Except for in the Ordos Plateau, a few *Y*.*pestis* isolates were collected not only in 2018 but also in 2019 in northern semidesert steppe areas. Such observations indicated that plague epizootics had already existed in these areas in 2018 and were more violent in 2019. Such a continuous occurrence pattern was also previously observed in other endemic areas, for example, in Madagascar[15].

Even *Y*.*pestis* strains in lineage 2.MED3m were major lineage generally, and this lineage affected most of the geographical area in the Inner Mongolia *M*.*unguiculatus* plague focus in 2018 and 2019, one of interesting observations is that we found that these rodent plagues in different areas in Inner Mongolia in 2019 belonged to different epizootics (three different lineages, i.e., 2.MED3m, 2.MED3q, 2.MED3r) rather than being caused through the spreading of the same *M*.*unguiculatus* epizootic (S1 Fig), even though the areas all belong to the *M*.*unguiculatus* plague focus. Another interesting observation is that one of the previously isolated strains (Shanxi 11, isolated from adjacent Shanxi Province in 2006, also belong to *M*.*unguiculatus* plague focus) also clustered in 2.MED3m, which indicates that such lineages of *Y*.*pestis* had continuously existed in this focus.

Because no geographic barriers existed in the northern desert steppe area on the Inner Mongolia Plateau, the epizootics were associated with lineage 2.MED3m was more intensely prevalent, not only in the temporal dimension but also in the spatial dimension. In addition, even though the Yellow River, as an obvious geographical barrier, separates the Inner Mongolia Plateau into the Ordos plateau and northern semidesert steppe areas, the strains of lineage 2.MED3q was found on both sides of the Yellow River (S1 Fig).

## Discussion

Many factors, including those of biotic (ecological) and abiotic origin (environmental: humidity, soil chemistry, etc.), influence *Y*.*pestis* and animal plague dynamics [16]. In fact, any ecological disturbance that changes the dynamics of the main host or main vector, including weather changes, or even human activities such as agricultural activities or mass killing rodents by rodenticides, could unleash a flood of homeless fleas. When these fleas are transferred to humans or domestic animals, these fleas could cause hosts to become infected. According to previous observations, plague epizootics in Inner Mongolia Plateau *M*.*unguiculatus* plague foci could prevail all year, and two peaks of prevalence were observed in April~May and October~November, even though the period of prevalence peak could last longer in certain years when violent epizootics were observed [4]. Previous studies provided insight into the relationship between plague intensity and the level of precipitation in the semiarid grasslands of Inner Mongolia [17], i.e., Plague epizootics depend on changes in the density and distribution of local *M*.*unguiculatus* [4], while elevated rainfall facilitates increasing population levels of *M*.*unguiculatus* by promoting seed production and growth of grass [17]. In 2019, additional rainfall stimulated the growth of grass, and these factors resulted in a great increase in the local rodent population in Inner Mongolia. This observation was consistent with previous research showing that precipitation could affect the intensity of *M*.*unguiculatus* plague epizootics [17].

In the four human plague cases in Inner Mongolia and in Beijing, only one *Y*.*pestis* isolate (from patient C) was successfully cultured. The other three patients all had no positive culture results. Therefore, the diagnosis had to be based on immunological and PCR methods. Failures of culture were due to the usage of antibiotics[18]. For example, patients A and B, before the local professionals in the CDC tested their clinical specimens to confirm *Y*.*pestis* infection, nine or five days had passed, and a large amount of antibiotics were used during this period, so the loads of bacteria in the patients’ bodies were very low. This was the reason that no culture-based bacterial evidence was obtained by local CDC.

In epidemiologic investigations, the use of reliable subtyping methods is a prerequisite for the identification of the links among isolates and for understanding the dynamics of pathogen spread. Many molecular genotyping methods have been developed to illustrate the genetic relationships of *Y*.*pestis*[19], such as genome-based SNP analysis with the application of genome sequencing, clustered regularly interspaced short palindromic repeats (CRISPR), different region analysis (DFR), and MLVA[20, 21]. Genome-wide SNP analysis could be used to illustrate the phylogenetic relationship or the microevolution of strains, including for source-tracking purposes in outbreaks [6, 22]. When the pathogens could not be obtained by culture from clinical specimens or the genomic sequencing data were not enough to establish a phylogenetic tree, PCR-based molecular methods, such as the MLVA14 scheme or canonical SNP analysis, could be used to illustrate corresponding phylogenetic relationships [23] in plague outbreaks. In this study, seven SNP locus markers were used to further differentiate patient-associated pathogen DNA and classified them into corresponding lineages in the 2.MED3 population.

The Inner Mongolia Province in China borders Mongolia and Russia, and it is also adjacent to nine provinces in China. There were no obvious geographic barriers between the *M*.*unguiculatus* plague focus in Inner Mongolia and its counterpart area in Mongolia. Because convenient transportation and flourishing global trades could facilitate the rapid spread of infectious diseases worldwide, national health services in adjacent countries should work together to better identify the risk of human plague and animal plague, including establishing communication channels to exchange information about the occurrence of human plague cases and rodent surveillance data, providing early warnings bilaterally, carrying out joint animal plague investigations, and jointly controlling epizootics in border areas when necessary.

## Supporting information

S1 Table *Y.pestis* strains used to establish the phylogenetic relationship in this study. (XLS)

S2 Table List of SNPs in 2.MED3 population for construction of MSTree. (XLSX)

S3 Table Seven SNP loci used to differentiate Inner Mongolia strains in the 2.MED3 and corresponding primers for PCR. (DOCX)

S4 Table Profiles of 14 VNTRs and alleles of seven SNPs used for identifying the position of phylogenetic lineages of DNAs or strains isolated in Inner Mongolia in 2018 and 2019. (XLSX)

S1 Fig Geographical distribution of isolates in different epizootics in Inner Mongolia in 2018 and 2019. The base layer of the geographic background map sourced from an open maps access (https://eol.jsc.nasa.gov/SearchPhotos/). The nomenclature of natural plague foci in China is consistent with a previous report [24].
(TIF)
