# Supplementary material for: Genetic source tracking of human plague cases in Inner Mongolia-Beijing, 2019
Source: PLoS Negl Trop Dis. 2021 Aug 3;15(8):e0009558. doi: 10.1371/journal.pntd.0009558 (PMC8362994; doi:10.1371/journal.pntd.0009558)
Supplement: S3 Table — (DOCX) [file pntd.0009558.s003.docx]

S3 Table. The seven SNP loci used to differentiate Inner Mongolia strains in 2.MED3 lineage and their primers for PCR

| Code | Name of Primer | **Nucleotide Sequence (5’ to 3’)** | **Product Size** | **SNP ID #** | **Position in *Y.pestis*** CO92 | MSTree Branch | Anc Nt- Der Nt |
| --- | --- | --- | --- | --- | --- | --- | --- |
| 1 | s217-F  s217-R | GATCAGAGCCAACTGAGGCA  ACAGACGCCCGCATTATACC | 472 | s217 | 480174 | N50_2.MED3k | A-G |
| 2 | s2563-F  s2563-R | TGGTGTCAGAATGGTGGTGG  GCATCATACCCAAGGGGACA | 494 | s2563 | 423172 | N50_2.MED3k | C-T |
| 3 | s2599-F  s2599-R | AACATAACCTGCAACGCGGA  TCTCCCAAGCATGGAGTTTATGA | 463 | s2599 | 588771 | N54_2.MED3l | G-T |
| 4 | s2876-F  s2876-R | GGGGGTTTTTATTTCACGTCAGG  GGTCCCTTCATCCAGCACAT | 531 | s2876 | 2320808 | N50_2.MED3k | G-A |
| 5 | s3032-F  s3032-R | ACGCATAAACCACACCGTCA  ACTTCCGTATTTGCTGCCGA | 577 | s3032 | 3212272 | N50_2.MED3k | C-T |
| 6 | s3244-F  s3244-R | AAGGTCAGTACGAAGGGGCT  TGTGGATTGCGGTGGGGATA | 529 | s3244 | 4326677 | N54_2.MED3m | A-C |
| 7 | s3387-F  s3387-R | GCCCAATACGGCACTCAAAT  CGGTTGGGCGTAACAGTGAA | 485 | s3387 | 979204 | 2.MED3k_N54 | A-G |

**#** **name of SNPs used in construction of MSTree cited from previous literature:** Cui Y, Yu C, Yan Y, Li D, Li Y, Jombart T, Weinert LA, Wang Z, Guo Z, Xu L,Zhang Y, Zheng H, Qin N, Xiao X, Wu M, Wang X, Zhou D, Qi Z, Du Z, Wu H, Yang X, Cao H, Wang H, Wang J, Yao S, Rakin A, Li Y, Falush D, Balloux F, Achtman M, Song Y, Wang J, Yang R. Historical variations in mutation rate in an epidemic pathogen, *Yersinia pestis*. Proc Natl Acad Sci U S A. 2013 Jan 8;110(2):577-82.doi: 10.1073/pnas.1205750110. Epub 2012 Dec 27. PubMed PMID: 23271803; PubMed Central PMCID: PMC3545753.

Anc: ancestral; Der - derived; Nt: Nucleotide;
