# Supplementary figures and images for: Genetic source tracking of human plague cases in Inner Mongolia-Beijing, 2019
Source: PLoS Negl Trop Dis. 2021 Aug 3;15(8):e0009558. doi: 10.1371/journal.pntd.0009558 (PMC8362994; doi:10.1371/journal.pntd.0009558)

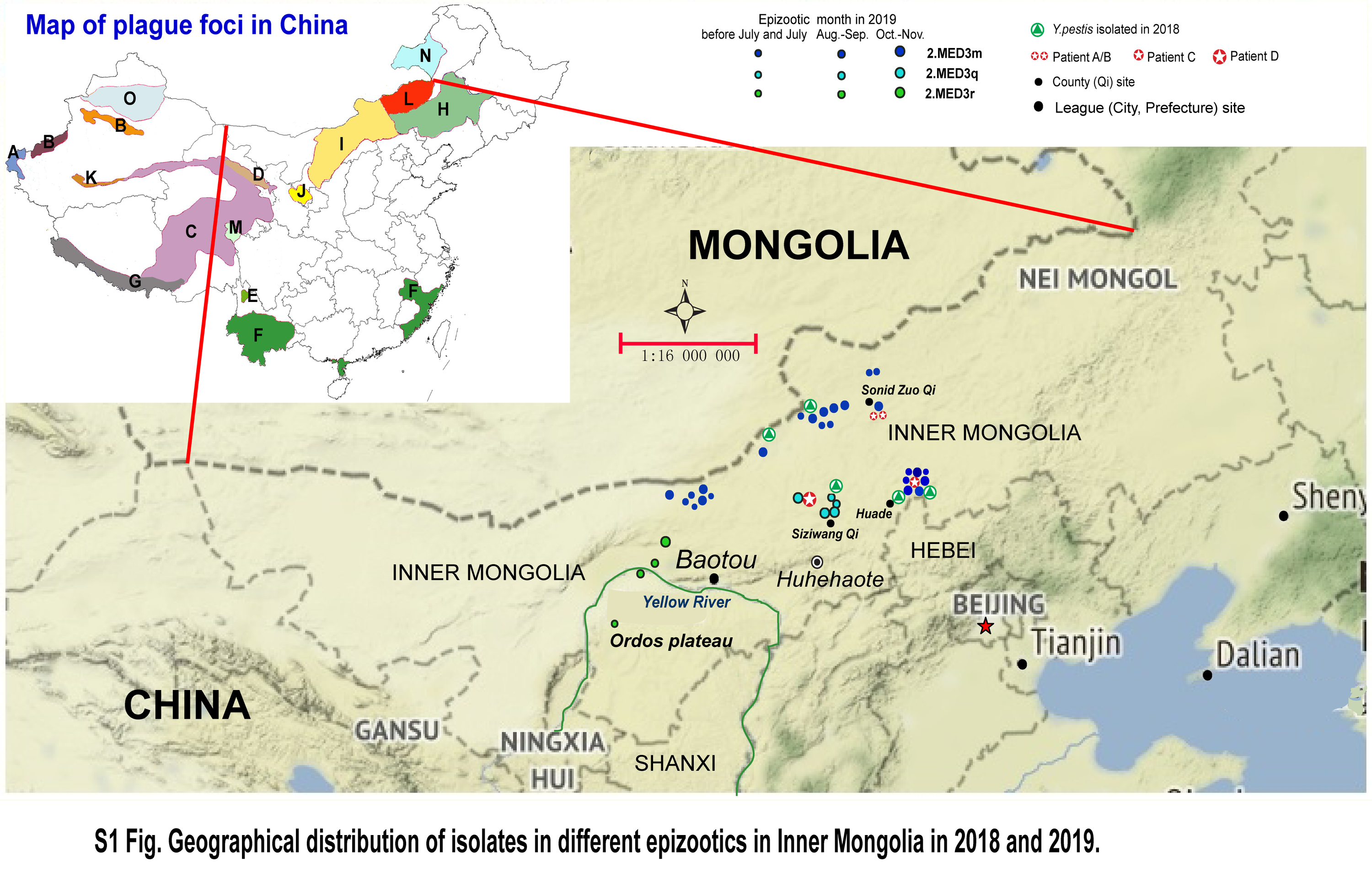

Supplement: S1 Fig — The base layer of the geographic background map sourced from an open maps access (https://eol.jsc.nasa.gov/SearchPhotos/). The nomenclature of natural plague foci in China is consistent with a previous report [24]. (TIF) [file pntd.0009558.s005.tif]
